# Bitter gourd has the highest azoxystrobinon residue after open field application on four cucurbit vegetables

**DOI:** 10.1371/journal.pone.0203967

**Published:** 2018-10-31

**Authors:** Gang Guo, Fengmao Liu, Yanli Bian, Xiaohan Li

**Affiliations:** College of Science, China Agricultural University, Beijing, China; Chinese Academy of Agricultural Sciences Institute of Plant Protection, CHINA

## Abstract

The goal of this study was to select a representative cucurbit vegetable crop that contained the highest residue levels of the pesticide azoxystrobinon. To do this, we used open field application of azoxystrobinon in four cucurbit crops (cucumber, zucchini, bitter gourd, and loofah) in Beijing, Shandong, and Anhui. Liquid chromatograph-mass spectrometry/mass spectrometry (LC-MS/MS) with selected reaction monitoring was used to determine azoxystrobinon levels in each of the selected cucurbit vegetables. The azoxystrobinon limit of detection was 0.005 mg kg^-1^ for all samples. Recoveries of azoxystrobinon ranged from 94.2% to 107.1% at spiked levels of 0.005–0.5 mg kg^-1^. In field trials, the half-life of azoxystrobinon in each of the four cucurbit crops was within the range of 1.4–3.1 d. Based on these results, we recommend that bitter gourd is selected as a representative cucurbit vegetable for future studies of azoxystrobinon. The obtained residual data were also assessed for their dietary risk and results indicated that there is no chronic dietary risk in any of the four, selected cucurbit vegetables. The recommended maximum residue limit (MRL) of azoxystrobinon in this subgroup was 0.2 mg/kg.

## 1. Introduction

Cucumber *(Cucumis sativus L*.), zucchini *(Cucurbita pepo Linn)*, bitter gourd *(Momordica charantia L*.*)*, and loofah *(Luffa cylindrica (L*.*) Roem*.*)* are cultivated in many parts of the world and are especially prevalent in China. Despite their prevalence, zucchini, bitter gourd, and loofah are all regarded as minor crops. In most parts of world, these four crops account for a relatively small area of all crops cultivated. They typically form many small, specialty crops that cover a small and scattered cultivating area and use a limited amount of pesticides. Given these metrics, pesticide manufacturers are not willing to spend the time and money to register their products for such crops and therefore there are relatively few pesticide products registered for such small crops.

These problems are widely seen across the world. The EU and US along with the Codex Alimentarius Commission (CAC) established crop classifications that contain delineated groups and subgroups [1–3]. In 2006, the CAC’s Crop Classification Revision program began. Since then, crop grouping and group representative crops have been under discussions for 11 years and the work is not yet completed. This is not surprising, since there is a large amount of information and the task is complicated. Moreover, there is considerable controversy when selecting representative crops and formulating group MRLs. It is also worth noting that this classification—when completed—will enable small crops to use pesticides more scientifically and rationally.

The CAC divides crops into groups and subgroups and then selects representative crops from each subgroup. The representative crops are typically those with the highest residue or the most economic value in each groupand are used to determine suitableMRLs for the whole group. Currently, Group 011 Cucurbit Fruiting Vegetables is the CAC classification’s biggest challenge. This group crop classification has been discussed for over four years, from the 45^th^ International Commission on Pesticide Residues in the Codex Alimentarius (CCPR) to the 48^th^ CCPR conference [3–6]. In 2015, the 47^th^ CCPR discussedsubgroup 011 of the Cucurbit Fruiting Vegetables group. From these discussions, three options were proposed: Option 1 (three subgroups, supporting comments from Thailand and Japan): Subgroup 11A Cucumber and Summer Squash, Subgroup 11B Melons, and Subgroup 11C Winter squashes; Option 2 (two subgroups, International Crop Grouping Consulting Committee, supporting comments from Canada and the US): Subgroup 11A Melons and Subgroup 11B Squash/Cucumber subgroup; Option 3 (two subgroups, supporting comments from New Zealand): Subgroup 11A Cucurbits with Edible Peels and Subgroup 11B Cucurbits with Inedible Peels.

The main reason for the lack of consensus is that many cucurbit crops do not have relevant residual data. It took until the 49^th^ CCPR to make a final decision regarding the Cucurbit Fruiting Vegetables, which was as follows: 011A Fruiting vegetables, Cucurbits (cucumbers and summer squashes) and 011B Fruiting vegetables, Cucurbits (melons, pumpkins, and winter squashes) [7]. This final agreement took several rounds of discussion because most countries and regions have no related residue data; particularly for zucchini, loofah, and bitter gourd.

Azoxystrobin, methyl (E)-2-{2[6-(2-cyanophenoxy) pyrimidin-4-yloxy] phenyl}-3-methoxyacrylate, is a new type of efficient, broad-spectrum, systemic fungicide [8]. Strobilurins were first discovered from rotted Basidiomycetes [9]. Azoxystrobin, in particular, can be used for a variety of applications, including in stem and leaf sprays, seed treatments, and even be used to treat the soil itself. The mechanism of action of azoxystrobin is the blocking the bacteria c1 complex [10,11]. Azoxystrobin is similar to other strobilurins and has been shown to both increase the production and improve the quality of agricultural products [12,13].

Azoxystrobin and similar pesticides act to reduce post-harvest deterioration through anti-aging and water-saving physiological changes in the vegetables [14–17]. At present, there are few studies that have determined azoxystrobin levels in cucumber, as most studies assessing azoxystrobin have been done in cereal crops [17, 18]. In previous studies, wheat and maize treated with azoxystrobin showed significant yield increases [18, 19]. Moreover, no studies have determined azoxystrobin levels in cucurbit vegetables as a whole—especially in bitter gourd, loofah, and zucchini.

This lack of information on azoxystrobin in cucurbit vegetables is concerning, given that it is used on many fruits and vegetables. While some papers have focused on the dietary risk of azoxystrobin, there has been little to no discussion on individual MRLs or group MRLs [20].

The main focus of the work presented here was to establish the MRLs in cucurbit vegetables. This was done to not only reduce pesticide exposure in humans, but also to provide a database for the crop classification of cucurbit vegetables. The Chinese classification of these crops includes the following categories: (a) cucumber and gherkins, (b) zucchini, bitter gourd, loofah, and (c) wax gourd and pumpkin [21]. The European Union’s classification of fruiting vegetables is as follows: (a) Solanaceae and Malvaceae, (b) cucurbits with edible peel, and (c) cucurbits with inedible peel [1]. In the US, the classification is as follows: (a) melons subgroup and (b) squash /cucumber subgroup [2].

## 2. Materials and methods

### 2.1 Reagents and materials

The azoxystrobin standards (98.5%) were purchased from the Agricultural Environmental Protection Institution of Tianjin, China. Azoxystrobin (SD; 250g/L) was purchased from Syngenta, China. The analytical reagents acetonitrile and methanol were obtained from Burdick & Jackson, USA. Ultra-pure water was purified using a Milli-Q water purification system (ThermoFisher Scientific, USA).

A stock solution of azoxystrobin (1000 mg L^-1^) was prepared in HPLC-grade acetonitrile. Cucumber, zucchini, bitter gourd, and loofah were cultivated in Beijing, Shandong, and Anhui.

### 2.2 Instrumentation

An Agilent 1200 Series Liquid Chromatograph was coupled with a 3200 QTRAP Mass Spectrometer. Briefly, analyses were separated using an Agilent C18 column (50 mm × 2.1 mm, 2.1 μm, 100 Å), the column temperature was set to 25°C, and the analytical column was equipped with a 2 mm ID C18 pre-column. Aqueous solutions of formic acid (0.1%) and methanol were used as mobile phase components A and B, respectively. The mobile phase was set at a flow rate of 0.2 ml/min in isocratic elution mode: 0–3 min 90% B.

The mass spectrometer was operated in the selected reaction monitoring (SRM) mode. Mass spectra in total ion current (TIC) mode were also acquired across a range of 100–600 m/z. Optimized electrospray ionization (ESI) conditions were as follows: Source temperature 450°C, nitrogen used as curtain (3 × 105 Pa) and auxiliary gas (3 × 105 Pa), and collision gas (He). Mass spectra (TIC and SRM modes) were acquired in the positive ionization mode and the capillary voltage was −4.5 kV and + 5.5 kV, respectively. The collision energy for each monitored transition was optimized in SRM mode. The monitored azoxystrobin transitions were m/z 404.0/344.1 at 55 V and 404.0/372.1 at 25 V. The SRM mode of the degradation patterns m/z 404.0/372.1 was used for quantification, and both 404.0/344.1 and 404.0/372.1 were used for confirmation. MS conditions were optimized by injecting an azoxystrobin standard (5 μL; 20 μg/mL into the sample matrices.

### 2.3 Experimental design of residue field trials

The field experiments using azoxystrobin were conducted on cucumber, bitter gourd, zucchini, and loofah. All four vegetables were grown in Beijing, Shandong, and Anhui on cropping areas determined to be free of azoxystrobin prior to the start of the study.

**Fig 1:**
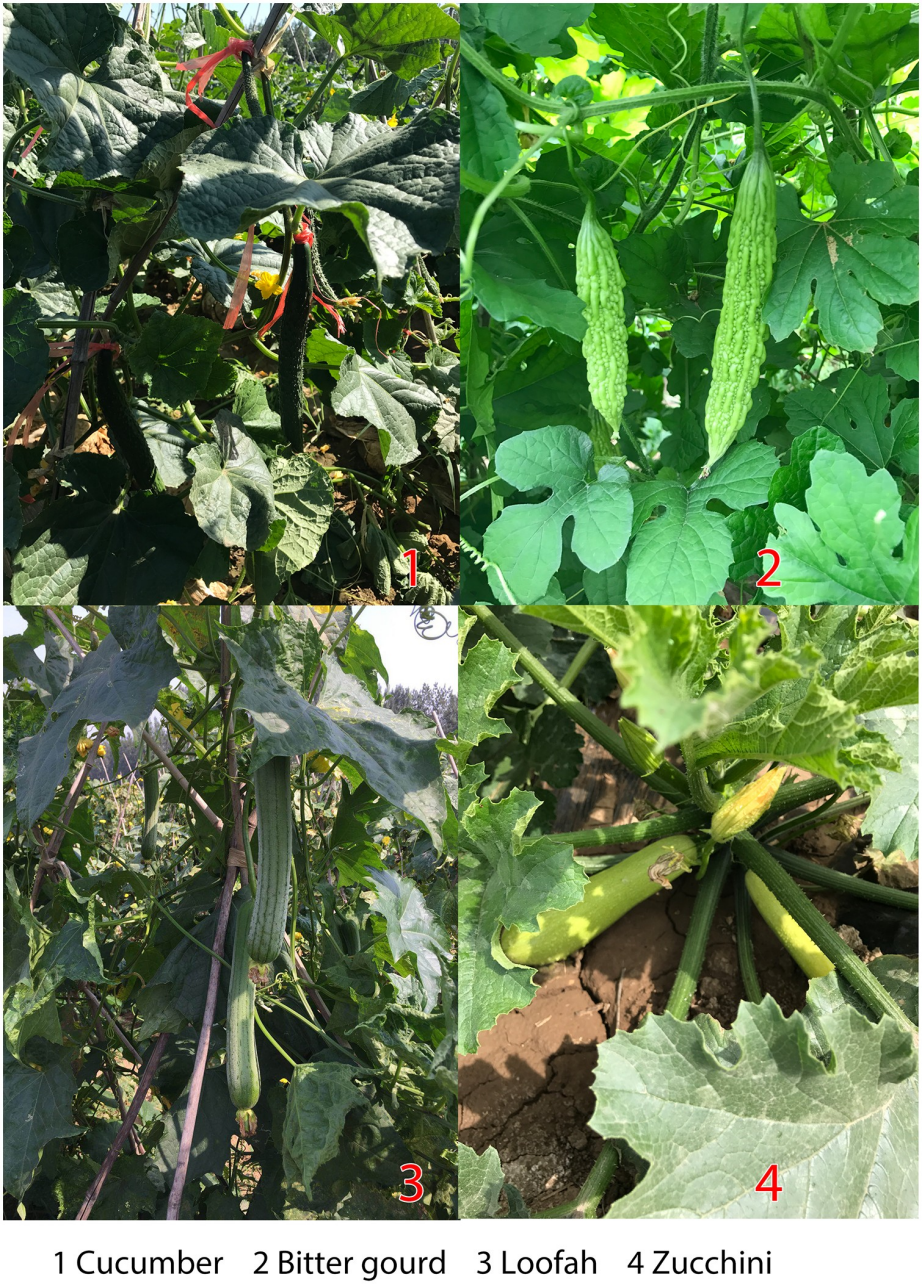
Representative field pictures of cucumber, bitter gourd, loofah, and zucchini.

In accordance with the “Guideline on Pesticide Residue Trials” issued by the Ministry of Agriculture of the People’s Republic of China (Standard Operating Procedures on Pesticide Registration Residue Field Trials, 2018), all field experiments were carried out with the recommended dosage of 337.5 g ai ha^-1^ of azoxystrobin. A 250 g/L SD formulation was sprayed for a total of three times, within an interval of 7 days. Each treatment area was composed of three replicate plots and one control plot. Samples were collected at several times: 2 h, 2 days, 5 days, 10 days, 14 days, and 21 days after one treatment with azoxystrobin. Representative cucumber, bitter gourd, zucchini, and loofah samples were randomly collected from each plot after the azoxystrobin application. Representative images of these four crops can be seen in Fig 1. All samples were individually stored in polyethylene boxes at -20°C until later analysis. This storage method was chosen to avoid any sample degradation between sampling and analysis.

### 2.4 Sample preparation

**Fig 2:**
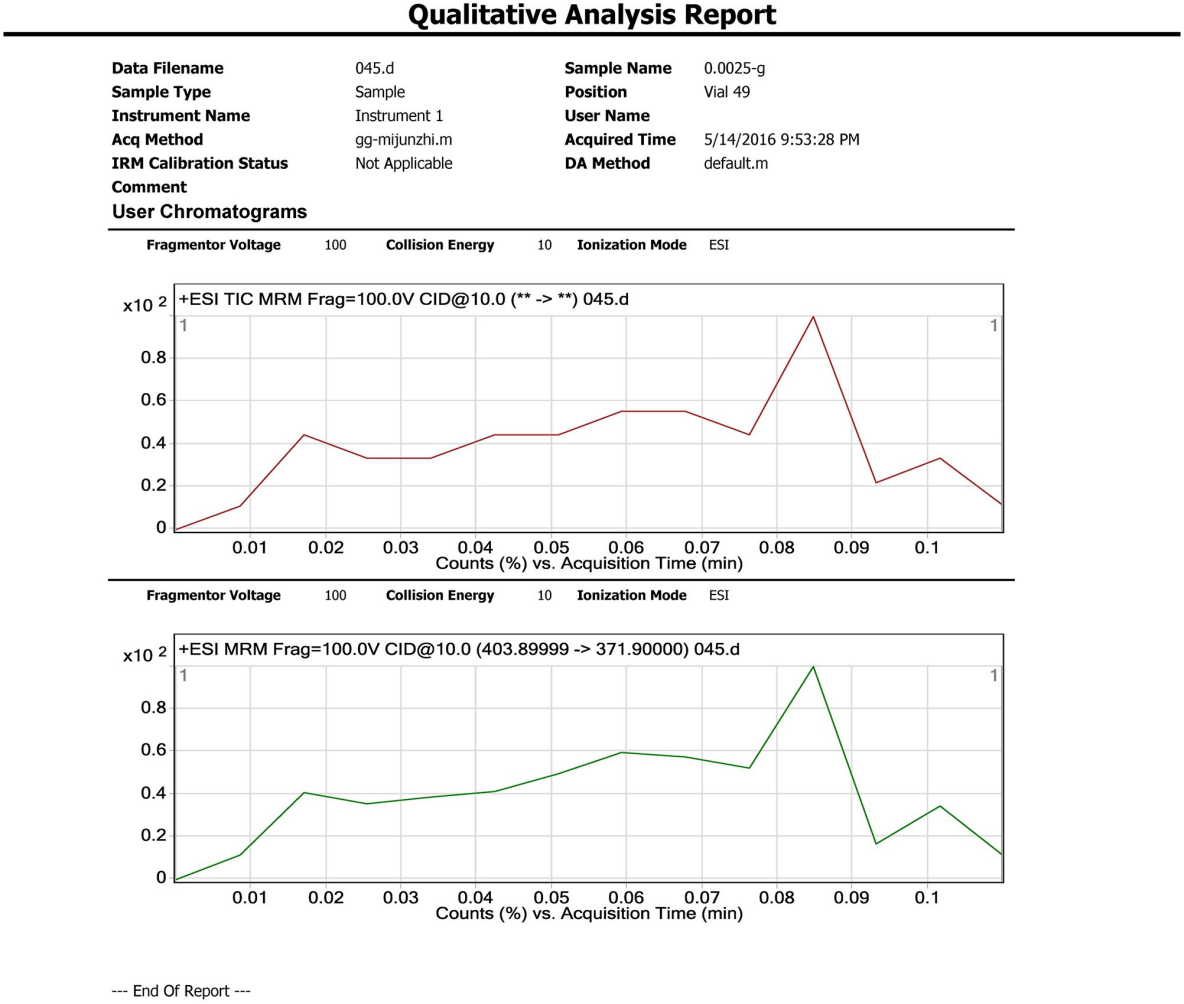
Cucumber CK.

**Fig 3:**
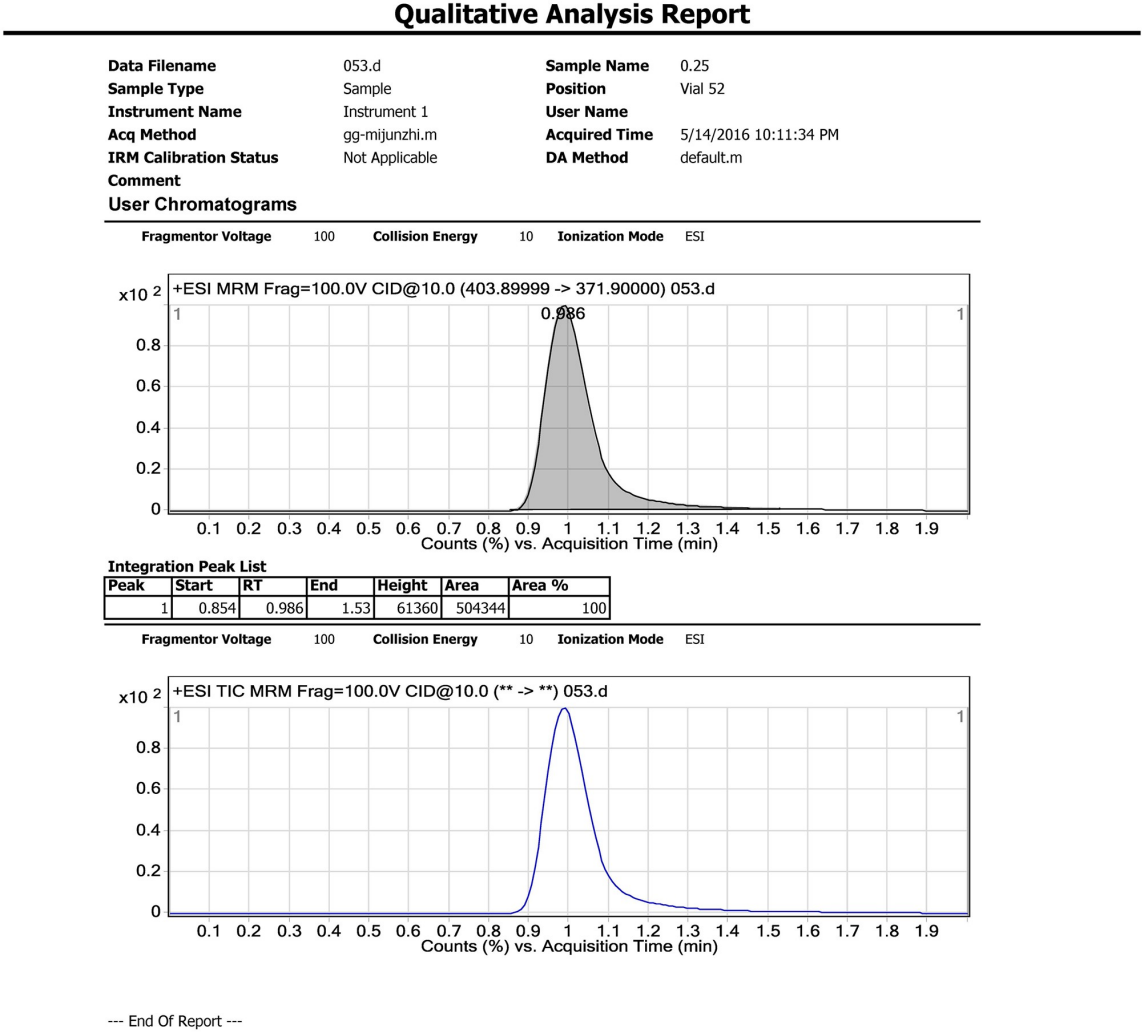
Matrix standards (0.25 mg/kg).

**Fig 4:**
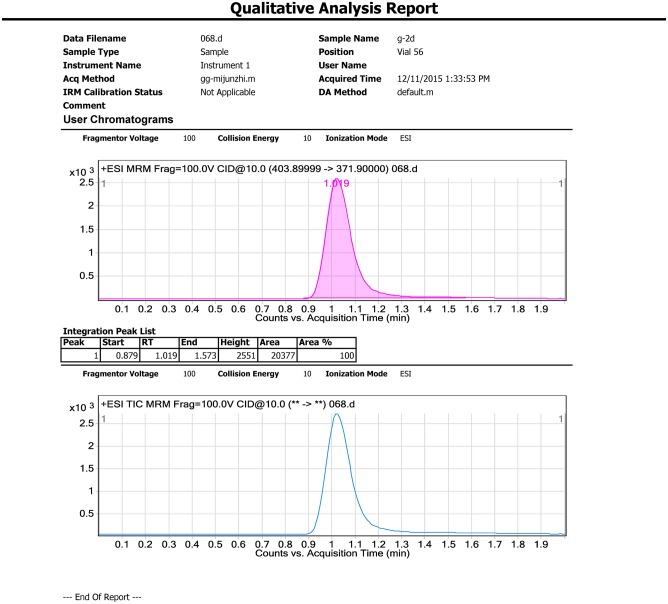
Representative samples.

Samples (5 g) were individually placed into 50 mL Teflon centrifuge tubes. 20mL MeCN, 4 g MgSO_4_, and 1 g NaCl were added to each sample and tubes were vortexed for 1 min. The mixture was then centrifuged for 5 min at 3800 rpm. Each supernatant (1 mL) was transferred to a 2 mL microcentrifuge tube containing 20 mg Primary-secondary amine (PSA) sorbent and 10 mg of purifying graphitized carbon black (GCB). The tubes were vortexed again for 1 min and centrifuged for 1 min at 10000 rpm. The purified supernatant was filtered using a polytetrafluoroethylene (PTFE) membrane filter (0.22 μm, Agela^®^, China) and 1.0 mL extract was analyzed using LC-MS/MS. Representative chromatograms are shown in Figs 2–4.

## 3. Result and discussion

### 3.1 Method validation

The identification and quantification of azoxystrobin was based on high- performance liquid chromatography using electrospray ionization-tandem mass spectrometry retention periods. Peak areas were compared against standard calibration curves. The limits of detection (LODs) and the limits of quantitation (LOQs) for azoxystrobin were taken as the concentrations that produced at a signal-to-noise (S/N) ratio of 3 and 10, respectively. In this study, the LOD for azoxystrobin was 0.001 mg kg^-1^ and the LOQ was 0.003mg kg^-1^. The matrix standard curve is shown in S1 Table.

The four vegetables were prepared using a modified QuEChERS method. Accuracy was determined using the spike recoveries at various levels in a complex matrix. The azoxystrobin recoveries for the four vegetables were all within 92.4%–112.1% at concentrations are 0.005, 0.05, and 1.0 mg kg^-1^. The results are shown in S2 Table.

### 3.2 Dissipation curve

**Fig 5:**
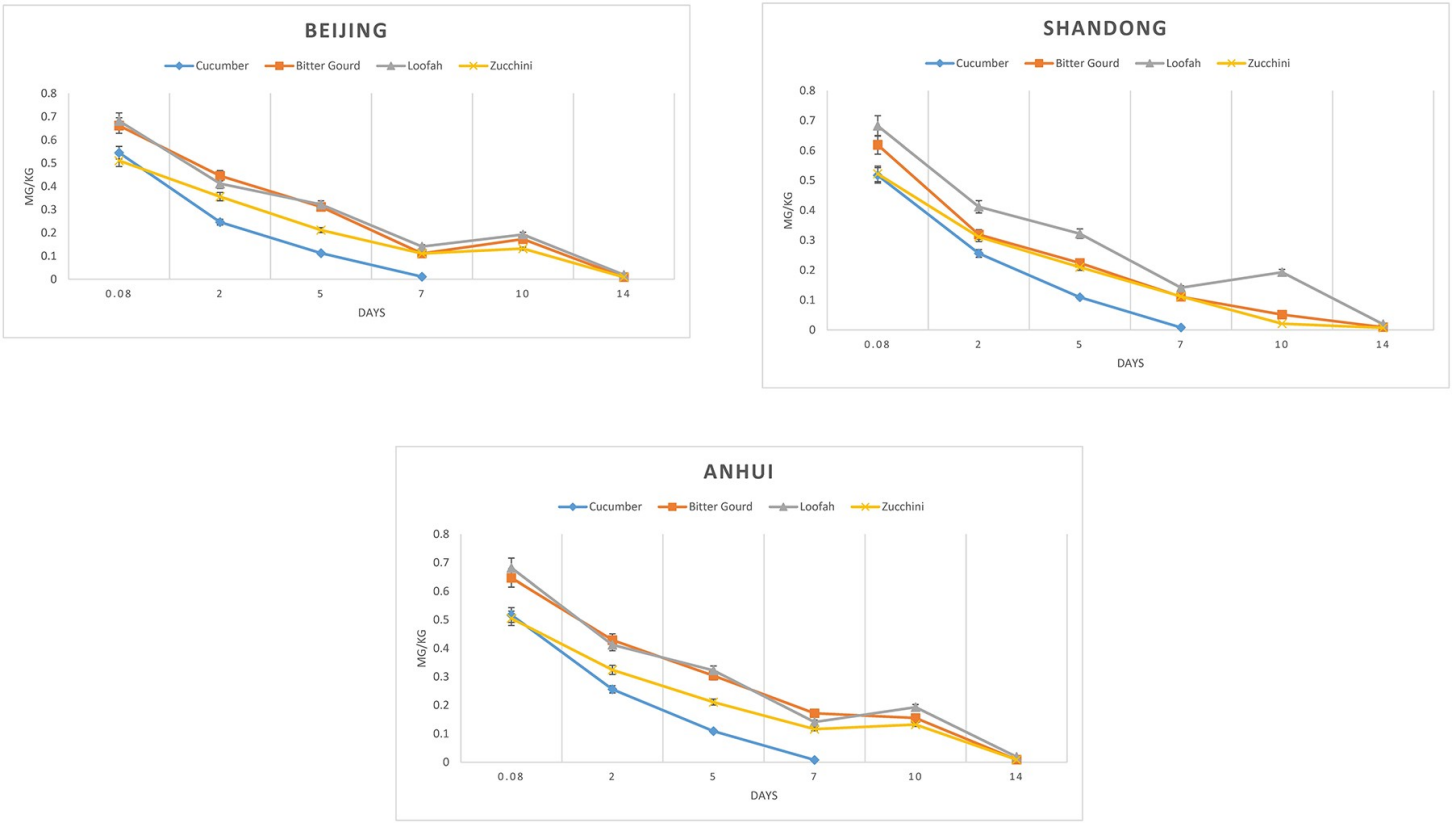
Dissipation curve of azoxystrobin in the four cucurbit fruiting vegetables grown in Beijing, Shandong, and Anhui.

The azoxystrobin dissipation curve for the four cucurbit fruiting vegetables are listed in Fig 5. The dissipation process complied with a first-order kinetic reaction. The first order rate equation was used to calculate the degradation rate and half-life using the following equation: C_t_ = C_o_e^-kt^, where Ct represents the concentration of pesticide residues at time t, C_o_ represents the maximum concentration after pesticide application, and k is the dissipation degradation rate constant (day^-1^). The half-life (t_1 / 2_) was calculated from the k value of each experiment (t_1 / 2_ = ln2 / k). C_t_ = C_o_e^-kt^, the half-life and R^2^ of the residue dissipation, are summarized in S3 Table.

The initial azoxystrobin residue was between 0.505–0.731 mg/kg and the half-life was 1.4–3.7 days. The coefficient of determination was 0.834–0.942. According to China’s current crop classification, cucumber and several other cucurbit fruiting vegetables are not included in the same crop group. However, considering the half-lives and initial azoxystrobin residues, it is reasonable to classify them in the same group. Recently, the CAC spent four years discussing the classification of cucurbit fruiting vegetables and whether to include them in the same group. The issue was finally resolved at the 49^th^ CCPR. At present, nectarine and peach are in discussion to be included in the same crop group. However, the 100g/L WP of ethyl glutathione used in the Joint FAO/WHO Meeting on Pesticide Residues (JMPR) report is applicable to nectarines and peaches. In the case of dosage, the half-lives of peach is 11.2 days and the half-life of nectarine is 22.6. Initial residue in peach is approximately five times higher than that of nectarine. This difference can also be divided into the same group; given this, peach can be chosen as a representative crop when extrapolating CAC crop classification [22].

### 3.3 Terminal residues in field trials

**Fig 6:**
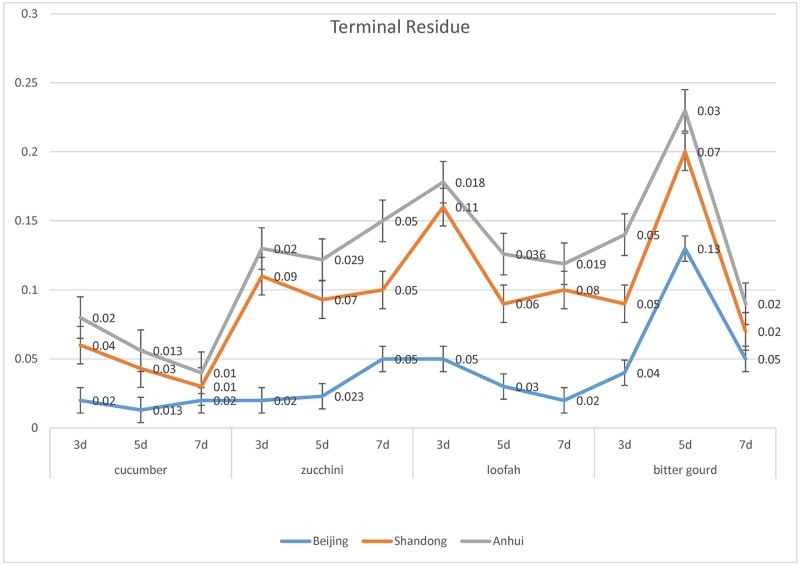
Terminal residues of azoxystrobin on PHI 3, 5 and 7.

The terminal azoxystrobin residues on PHI 3, 5, and 7 are shown in Fig 6. There was a slight difference in terminal residues between the three locations. Beijing experiments were conducted in May, while both Shandong and Anhui were conducted in June. The temperature and rainfall in Shandong and Anhui were higher than those of Beijing, Moreover, the average temperature in Anhui at that time was 2–3°C higher than Shandong. With these in mind, our results revealed that the terminal residues on samples from Anhui were the lowest.

When the PHI was three days, the lowest azoxystrobin STMR of the vegetables was cucumber (0.02 mg/kg) while the highest was bitter gourd (0.11 mg/kg). The lowest HR of the four vegetables was cucumber (0.05 mg/kg) and the highest was bitter gourd (0.12 mg/kg). When the PHI was five days, the lowest azoxystrobin STMR on the four vegetables was cucumber (0.014 mg/kg), while the highest was bitter gourd (0.06 mg/kg). The lowest HR of the four vegetables was cucumber (0.04 mg/kg) and the highest was bitter gourd (0.15 mg/kg). When the PHI was 7 days, the lowest azoxystrobin STMR on the four vegetables was cucumber (0.01 mg/kg), while the highest were both bitter gourd and zucchini (0.05 mg/kg). The lowest HR of the four vegetables was cucumber (0.02 mg/kg) and the highest was bitter gourd (0.09 mg/kg).

**Fig 7:**
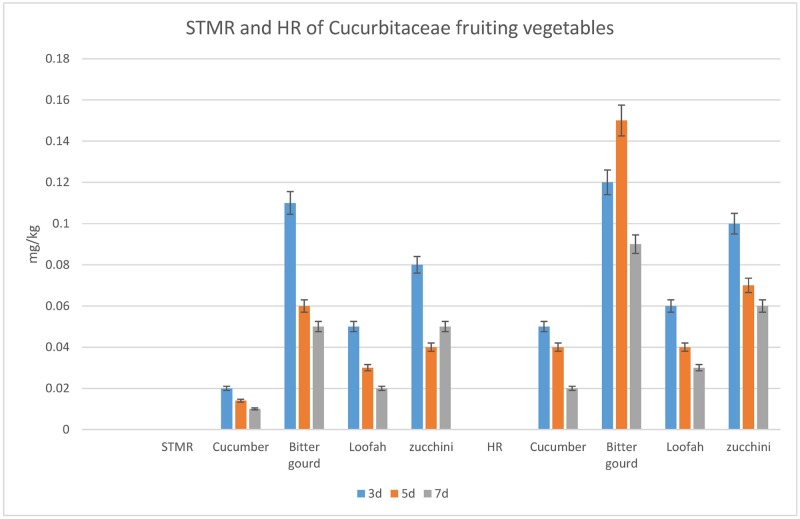
The STMR and HR of the tested cucurbit fruiting vegetables.

Given these results as well as the consideration of residual risk, bitter gourd can be used as a representative vegetable for the four cucurbit fruiting vegetables. In most countries, both regions and international organizations may choose the cucumber as a representative crop for use in trade and consumption. However, regions and international organizations must carefully set the MRLs, since the STMR of bitter gourd is 2.4–5.5 times higher than that of cucumber. The STMR and HR of cucurbit fruiting vegetables are shown in Fig 7. The STMR and HR of bitter gourd were higher than the other tested cucurbit fruiting vegetables. Given the CAC principle, bitter gourd may be used to represent the highest azoxystrobin on residue of the four cucurbit vegetables subjected to open field pesticide application. As per CAC principles, the same subgroup’s STMR needs to be less than five times to establish a subgroup MRLs. For example, sorghum and corn were initially in the same subgroup. However, comparison of the pesticide residue data revealed that they were in different subgroups: Subgroup 020D Grain Sorghum and Millet, Subgroup 020E Maize Cereals.

### 3.4 Health risk assessment

Public concern for pesticide residue risk has increased steadily; therefore, in this study, chronic and acute exposure risk assessment for different groups of people in China were investigated. These groups were chosen based on light-colored vegetable consumptions. After the chronic risk assessment, we used the average residue and STMR to establish the MRLs. Our dietary exposure assessment used both acute and chronic metrics. According to JMPR’s 2011 report, azoxystrobin’s acute reference dose (ARfD) is unnecessary because short-term intake does not pose a serious dietary risk to humans. Therefore, this study did not evaluate the acute exposure of azoxystrobin when found on melon vegetables.

The measures used for acute dietary exposure assessment were estimated short-term intake (ESTI) and ARfD. Chronic dietary exposure assessments were calculated based on estimated daily intake (EDI, mg kg^-1^, bw) and acceptable daily intake (ADI, mg kg^-1^, bw). They also accounted for the risk of the entire life-cycle. A risk quotient (RQc) > 100% indicated an unacceptable risk. The correlation equation used was RQc = EDI / ADI and a RQc <100% indicated no chronic dietary risk. The residual data we obtained were then used to establish corresponding group MRLs. The results are shown in S4 Table.

### 3.5 Subgroup MRLs

The method to determine the residue limits for the CAC crop groups was like that used for chlorantraniliprole (0.1 kg ai/ha, PHI 1day) [23]. The STMR of cucumber (n = 7) was 0.015 mg/kg while the STMR of melon (n = 7) was 0.065 mg/kg. The STMR of zucchiniresidue (n = 6) was 0.047 mg/kg. The residue ratio of melon/cucumber = 0.065/0.015 = 4.3 while the ratio of zucchini/cucumber = 0.047/0.015 = 3.1. JMPR uses the maximum STMR in field trials within crop groups to recommend crop group MRLs. In most cases, the STMR ratio is less than five for a given subgroup. [22]. Other methods used to establish the MRLs were the OECD and NAFTA calculators, which were as follows:

1. OECD: HR, Mean residue+4SD and CF×3 mean residue, where CF indicates correction factor, which is generally equal to 1.0. The OECD then used the largest of these as the recommended MRLs
2. NAFTA: Mean +3SD or 95/99th quantile rules were calculated as the MRL recommendation. For the ND or <LOQ residual value in the test data, the input was replaced by the LOQ value using the maximum likelihood estimate (MLE). When more than 60% of the residual data were unable to be accurately quantified, the NAFTA calculator should be used with caution. (Data above 20 points).

This paper uses the first two methods to calculate MRLs, using the largest of these as the recommended MRLs. The recommend MRL of azoxystrobin in the subgroup was determined to be 0.2 mg/kg. The MRL of azoxystrobin in cucumber of China was determined to be 0.5 mg/kg. The MRL of azoxystrobin in cucumber in the EU was determined to be 1.0 mg/kg. The MRL of azoxystrobin in cucurbits with edible peel in the EU was determined to be 1.0 mg/kg. The CAC and US do not have related MRLs and the different GAP (Good Agricultural Practices) directly impacts MRLs.

## 4. Conclusion

Based on both the planting area and consumption, we chose cucumber as our representation cucurbit crop. However, bitter gourd may have the highest azoxystrobinon residue after open field application of the four tested cucurbit crops. Given our findings, it is feasible to put cucumber, bitter gourd, loofah, and zucchini in the same subgroup. The half-lives of azoxystrobinon on the four cucurbit vegetables were within the range of 1.4–3.1 days. We found no chronic dietary risk of azoxystrobinon in these four cucurbit vegetables. The recommended MRLs of azoxystrobinon in this subgroup was found to be 0.2 mg/kg.

## Supporting information

S1 Table Standard curve equations and relative analyte parameters in Cucurbitaceae fruiting vegetables. (DOCX)

S2 Table Recoveries and relative standard deviations of azoxystrobin in Cucurbitaceae fruiting vegetables. (DOCX)

S3 Table Half-life and other statistical parameters for azoxystrobin in the four tested Cucurbitaceae fruiting vegetables. (DOCX)

S4 Table Chronic risk assessment of azoxystrobin in the four Cucurbitaceae fruiting vegetables. (DOCX)
