# Supplementary material for: Bitter gourd has the highest azoxystrobinon residue after open field application on four cucurbit vegetables
Source: PLoS One. 2018 Oct 31;13(10):e0203967. doi: 10.1371/journal.pone.0203967 (PMC6209134; doi:10.1371/journal.pone.0203967)
Supplement: S1 Table — (DOCX) [file pone.0203967.s001.docx]

**Table1 Standard curve equations and relative paraments of analytes in Cucurbitaceae vegetables**

| Pesticide | Matrix | Linear range（mg/L） | Linear equation | r | LOQ（mg/kg） |
| --- | --- | --- | --- | --- | --- |
| Azoxystrobin | Cucumber | 0.001～1 | y=1621158x+10074 | 0.9999 | 0.005 |
|  | Bitter gourd | 0.001～1 | y=177788x+22664 | 0.9994 | 0.005 |
|  | Loofah | 0.001～1 | y=1213442x-13226 | 0.9999 | 0.005 |
|  | Zucchini | 0.001～1 | y=271508.9x-7421 | 0.9995 | 0.005 |
