# Supplementary material for: Bitter gourd has the highest azoxystrobinon residue after open field application on four cucurbit vegetables
Source: PLoS One. 2018 Oct 31;13(10):e0203967. doi: 10.1371/journal.pone.0203967 (PMC6209134; doi:10.1371/journal.pone.0203967)
Supplement: S2 Table — (DOCX) [file pone.0203967.s002.docx]

**Table 2. Recoveries and relative standard deviations of Azoxystrobin in Cucurbitaceae fruiting vegetables**

| pesticide | Matrix | Concentrations（mg/kg） | Recovery（%） | | | | | Average Recovery（%） | RSD（%） |
| --- | --- | --- | --- | --- | --- | --- | --- | --- | --- |
| Azoxystrobin | Cucumber | 0.005 | 98.9 | 92.4 | 96.3 | 95.6 | 95.7 | 95.8 | 2.4 |
|  |  | 0.05 | 95.0 | 97.0 | 97.2 | 94.2 | 99.1 | 96.5 | 2.0 |
|  |  | 0.5 | 101.3 | 113.4 | 102.9 | 96.2 | 97.4 | 102.2 | 6.7 |
|  | Bitter Gourd | 0.005 | 96.7 | 107.1 | 101.8 | 105.4 | 104.5 | 103.1 | 3.9 |
|  |  | 0.05 | 96.9 | 108.3 | 93.8 | 94.9 | 101.1 | 99.0 | 6.0 |
|  |  | 0.5 | 96.6 | 112.1 | 108.9 | 99.5 | 104.2 | 104.3 | 6.1 |
|  | Loofah | 0.005 | 96.7 | 107.1 | 101.8 | 105.4 | 104.5 | 103.1 | 3.9 |
|  |  | 0.05 | 98.2 | 107.1 | 101.8 | 105.4 | 110.5 | 104.6 | 4.5 |
|  |  | 0.5 | 96.9 | 108.3 | 89.8 | 97.2 | 106.2 | 99.7 | 7.6 |
|  | Zucchini | 0.005 | 99.8 | 86.8 | 84.8 | 102.0 | 102.5 | 95.2 | 8.6 |
|  |  | 0.05 | 96.9 | 94.5 | 90.7 | 107.1 | 91.8 | 96.2 | 6.8 |
|  |  | 0.5 | 97.3 | 103.4 | 94.2 | 101.5 | 99.5 | 99.2 | 3.6 |
