# Supplementary material for: Bitter gourd has the highest azoxystrobinon residue after open field application on four cucurbit vegetables
Source: PLoS One. 2018 Oct 31;13(10):e0203967. doi: 10.1371/journal.pone.0203967 (PMC6209134; doi:10.1371/journal.pone.0203967)
Supplement: S3 Table — (DOCX) [file pone.0203967.s003.docx]

**Table 3. Half-life and other statistical parameters for Azoxystrobin in four Azoxystrobin in four Cucurbitaceae fruiting vegetables**

| **pesticide** | **matrix** | **position** | **Regression**  **equation** | **Determination**  **Coefficient(R^2^)** | **Half-life（d）** | **C_0_（mg/kg）** |
| --- | --- | --- | --- | --- | --- | --- |
| **Azoxystrobin** | cucumber | Beijing | y = 0.694e^-0.194x^ | 0.937 | 3.6 | 0.694 |
|  | Bitter gourd | Beijing | y = 0.671e^-0.189x^ | 0.918 | 3.7 | 0.671 |
|  | loofah | Beijing | y = 0.654e^-0.205x^ | 0.942 | 3.4 | 0.654 |
|  | Zucchini | Beijing | y = 0.731e^-0.201x^ | 0.899 | 3.4 | 0.731 |
|  | cucumber | Shandong | y = 0.611e^-0.186x^ | 0.929 | 3.5 | 0.611 |
|  | Bitter gourd | Shandong | y = 0.624e^-0.187x^ | 0.891 | 3.7 | 0.624 |
|  | loofah | Shandong | y = 0.654e^-0.205x^ | 0.942 | 3.4 | 0.654 |
|  | Zucchini | Shandong | y = 0.622e^-0.202x^ | 0.901 | 3.4 | 0.622 |
|  | cucumber | Anhui | y = 0.517e^-0.492x^ | 0.857 | 1.4 | 0.517 |
|  | Bitter gourd | Anhui | y = 0.647e^-0.235x^ | 0.819 | 2.9 | 0.647 |
|  | loofah | Anhui | y = 0.662e^-0.207x^ | 0.842 | 3.1 | 0.662 |
|  | Zucchini | Anhui | y = 0.505e^-0.228x^ | 0.834 | 3.0 | 0.505 |
