# Supplementary material for: Bitter gourd has the highest azoxystrobinon residue after open field application on four cucurbit vegetables
Source: PLoS One. 2018 Oct 31;13(10):e0203967. doi: 10.1371/journal.pone.0203967 (PMC6209134; doi:10.1371/journal.pone.0203967)
Supplement: S4 Table — (DOCX) [file pone.0203967.s004.docx]

**Table 4. The Chronic risk assessment of Azoxystrobin in four Cucurbitaceae Fruiting Vegetables**

| **Matrix** | **PHI(d)** | **STMR(mg/kg)** | **EDI(μg/(kg bw•d))** | **RQ_c_(%)** |
| --- | --- | --- | --- | --- |
| **Cucumber** | 3 | 0.02 | 0.002 | 1 |
|  | 5 | 0.01 | 0.001 | 0.7 |
| **Bitter Gourd** | 3 | 0.11 | 0.011 | 5.5 |
|  | 5 | 0.06 | 0.006 | 3 |
| **Loofah** | 3 | 0.05 | 0.005 | 2.5 |
|  | 5 | 0.03 | 0.003 | 1.5 |
| **zucchini** | 3 | 0.08 | 0.008 | 4 |
|  | 5 | 0.04 | 0.004 | 2 |
